# Pentasystem embolism associated with transient antiphospholipid antibodies in severe M. *pneumoniae* pneumonia: a “Dual-Hit” case report and systematic review

**DOI:** 10.3389/fimmu.2026.1845246

**Published:** 2026-09-30

**Authors:** Yuhang Hu, Jian Chen, Lijuan Wu, Jing Xu, Xuemei Xu, Juan Du, Min Kang, Xianmin Wang

**Affiliations:** 1 Department of Pediatric Critical Care Medicine, Sichuan Provincial Maternity and Child Health Care Hospital, Chengdu, Sichuan, China; 2 Department of Radiology, Sichuan Provincial Maternity and Child Health Care Hospital, Chengdu, Sichuan, China

**Keywords:** antiphospholipid antibodies, dual-hit hypothesis, immunothrombosis, pentasystem embolism, severe *Mycoplasma pneumoniae* pneumonia (SMPP)

## Abstract

**Background:**

Thromboembolism is a critical complication of severe *Mycoplasma pneumoniae* pneumonia (SMPP) in children, yet its clinical spectrum and immunothrombotic mechanisms remain poorly defined. The “dual-hit” hypothesis—synergy between infection-induced inflammation and transient antiphospholipid antibodies (aPLs)—lacks integrated clinical validation.

**Methods:**

We report a case of extensive multi-system thrombosis in a 6-year-old boy with SMPP. A systematic review of literature (January 2020–December 2025) identified 19 studies comprising 199 pediatric cases.

**Results:**

In the index case, thrombosis coincided with hyperinflammation and transient aPLs, which seroconverted to negative upon recovery. Pooled analysis showed multi-system involvement in 27.1% of cases (54/199). Among tested patients (n = 65; 32.7% of the cohort), aPL positivity was 69.2% (45/65). Most patients achieved favorable outcomes with anticoagulation and immunomodulation.

**Conclusion:**

This study is consistent with the “dual-hit” model in SMPP, where systemic inflammation may prime the endothelium and transient aPL positivity coincides with an extensive thrombotic burden. The high prevalence of multi-system involvement supports low-threshold vascular imaging and aPLs screening, along with biomarker-guided management. Transient aPLs should be interpreted as a potential risk-amplifying biomarker rather than a proven causal driver of thrombosis.

## 1 Introduction

*Mycoplasma pneumoniae* (MP) is a predominant pathogen of pediatric community-acquired pneumonia (CAP), accounting for a substantial proportion of hospitalized cases in children (1–3). While traditionally viewed as a self-limiting respiratory infection, severe M. *pneumoniae* pneumonia (SMPP) is increasingly recognized as a systemic inflammatory disorder that frequently involves extrapulmonary sites (4, 5). Among its extrapulmonary complications, thrombotic events represent a critical phenotype with potentially devastating sequelae (2, 6, 7). While pulmonary embolism (PE) remains the most frequently documented manifestation (5, 6, 8–10), emerging evidence suggests that the thrombotic burden can extend well beyond the pulmonary vasculature.

Recent reports have sporadically identified isolated extrapulmonary emboli, including cerebral infarction (11, 12), intracardiac thrombus (13–15), and rare cases of mesenteric or splenic ischemia (16, 17). However, the simultaneous occurrence of multi-system embolism—defined as involvement of two or more distinct vascular beds—remains an exceptionally rare and challenging clinical entity. The pathophysiology underlying such extensive thrombotic burden is hypothesized to follow a “dual-hit” mechanism: an initial systemic inflammatory surge triggering endothelial injury, followed by an acquired immunothrombotic state, often evidenced by transient antiphospholipid antibodies (aPLs) or cold agglutinins (3, 18–21). Despite these hypotheses, a comprehensive synthesis of clinical characteristics and outcomes for this specific “multi-system” subgroup is lacking, as most existing literature focuses on single-site thrombosis.

This gap is clinically important: multi-system thrombosis in pediatric MPP involves multiple vital organs simultaneously and is associated with severe clinical manifestations and poor outcomes (2). These patients are also at risk of irreversible ischemic injury, prolonged intensive care, and potential long-term sequelae (6, 17). Early recognition is therefore critical, as a delayed diagnosis may postpone the initiation of anticoagulation and immunomodulation and allow the thrombotic burden to progress.

To address these gaps, we present the case of a 6-year-old boy with SMPP who developed concurrent emboli in five distinct vascular systems—pulmonary, cerebral, cardiac, renal, and peripheral arterial. To our knowledge, this represents the most extensive thrombotic burden reported in the pediatric literature to date, surpassing the three- or four-site involvement noted in previous severe cases (6, 17). Leveraging this index case alongside a systematic review of 199 related cases from 19 studies published between 2020 and 2025, we aim to: (1). provide clinical evidence consistent with the “dual-hit” immune-thrombotic model; (2). analyze the under-recognized prevalence of aPLs positivity in multi-system cases; (3). propose a stratified management strategy for early recognition and anticoagulation in this high-risk population.

## 2 Case description

### 2.1 Patient presentation and initial workup

A previously healthy 6-year-old boy was admitted to our Pediatric Intensive Care Unit (PICU) on December 20, 2025, following a 7-day history of refractory hyperpyrexia and severe cough. Prior management at a local facility, which included intravenous ceftriaxone (80 mg/kg/day) and azithromycin (10 mg/kg/day) for three days, failed to alleviate symptoms. Initial chest computed tomography (CT) at the local center had revealed consolidation in the right middle and both lower lobes, accompanied by pleural effusion and atelectasis.

### 2.2 Microbiological and immunological profiling

Upon admission, the patient exhibited a profound hyperinflammatory and hypercoagulable state. Critical laboratory values included leukocytosis (11.99 × 10^9^/L, 83.5% neutrophils), elevated C-reactive protein (CRP, 115.8 mg/L), Interleukin-6 (158.62 pg/mL), and Lactate Dehydrogenase (LDH, 1053 U/L). Notably, coagulation markers were substantially abnormal, with D-dimer levels of 14.06 mg/L and Fibrin Degradation Products (FDP) of 32.6 mg/L.

Etiological investigation confirmed severe *Mycoplasma pneumoniae* (MP) infection. Serology showed a high antibody titer (>1:2560). Targeted next-generation sequencing (tNGS) of respiratory specimens identified a high MP sequence load (43,716 reads), indicative of macrolide resistance, a finding corroborated by blood metagenomic NGS (789 reads) (**Supplementary Figure S1**; **Supplementary Table S1**). Screening for other common respiratory pathogens was negative.

### 2.3 Clinical progression: the “Pentasystem” thrombotic event

Despite escalated antimicrobial therapy (Meropenem and Doxycycline) and prophylactic anticoagulation (Enoxaparin 100 IU/kg/dose q12h), the patient’s condition deteriorated. On hospital day 2, the onset of left great toe pain prompted an urgent multi-modality vascular assessment, revealing a widespread multi-system thrombotic cascade. The anatomical distribution of these emboli is summarized in **Figure 1**, with detailed radiological findings provided in the **Supplementary Figures S2**–**S5**.

**Figure 1:**
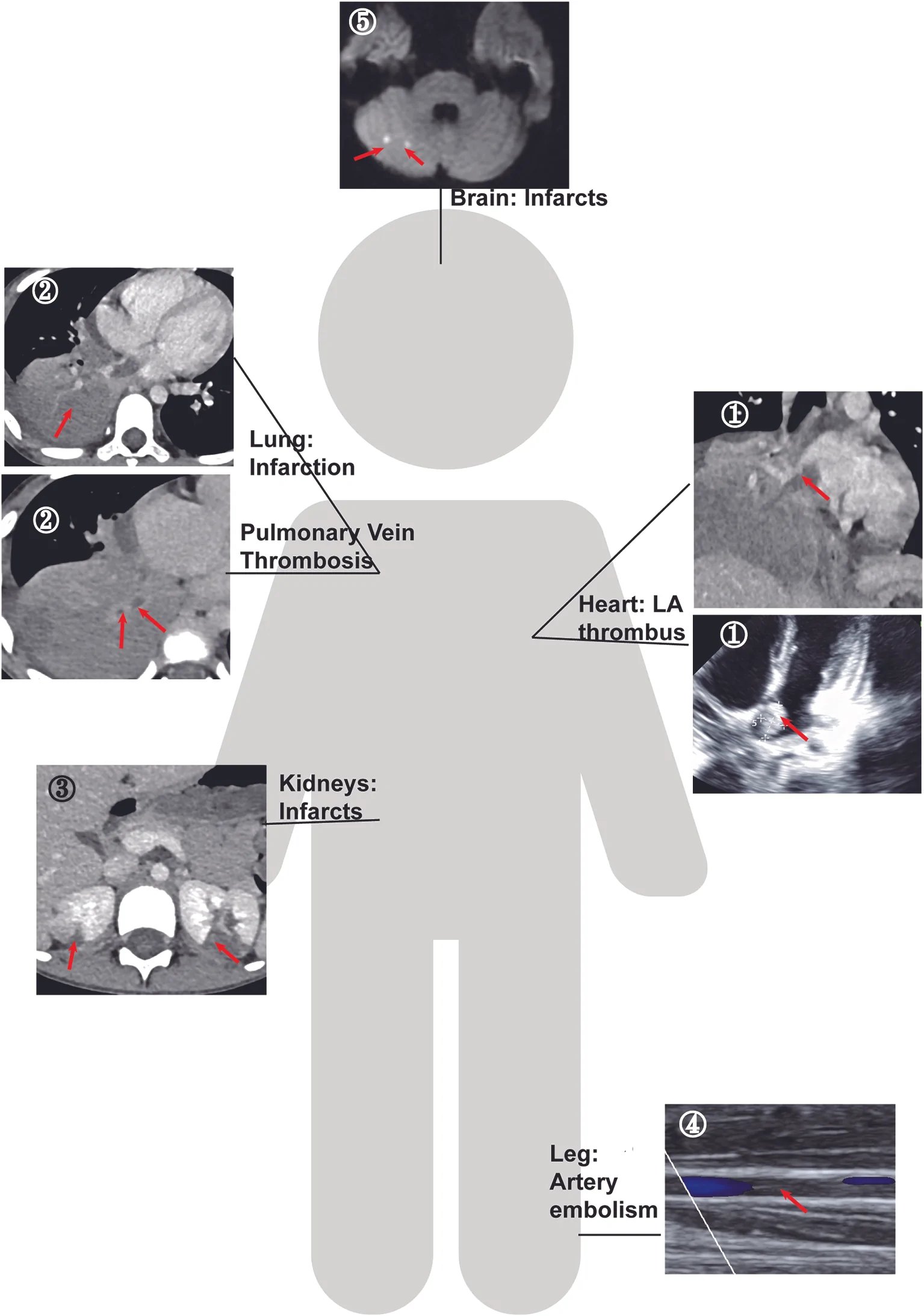
Anatomical mapping and radiological characterization of the “Pentasystem” thrombotic event. The central schematic illustrates the systemic distribution of emboli superimposed on a human contour. Surrounding panels display representative multi-modality imaging findings, with all pathological lesions indicated by red arrows: (1) Contrast-enhanced chest CT and transthoracic echocardiography revealing a large thrombus extending from the right inferior pulmonary vein into the left atrium; (2) Chest CT reconstruction showing segmental consolidation and infarction in the right lower lung lobe; (3) Abdominal CT demonstrating wedge-shaped hypodensities in bilateral kidneys consistent with renal infarction; (4) Lower limb Doppler ultrasound indicating absence of blood flow in the mid-to-distal segment of the left anterior tibial artery; and (5) Diffusion-weighted MRI (DWI) confirming acute ischemic lesions involving the left semioval center and right cerebellar hemisphere.

The thrombotic events involved five distinct vascular beds:

Cardio-Pulmonary: Contrast-enhanced chest CT and transthoracic echocardiography identified a large thrombus (approx. 1.7 × 1.1 cm) extending from the right inferior pulmonary vein into the left atrium (**Figure 1-(1)**; see also **Supplementary Figures S2**, **S3** for detailed multi-planar reconstruction).

Visceral Infarction: Concurrent segmental infarction in the right lower lung lobe and wedge-shaped infarctions in bilateral kidneys were documented (**Figures 1-(2), (3)**; **Supplementary Figure S2**).

Peripheral Vascular: Lower limb Doppler ultrasound confirmed embolic occlusion in the mid-to-distal segment of the left anterior tibial artery (**Figure 1-(4)**; **Supplementary Figure S3**).

Cerebral: On hospital day 3, brain MRI confirmed acute embolic infarcts involving the left semioval center, parieto-occipital white matter, and right cerebellar hemisphere (**Figure 1-(5)**; **Supplementary Figure S4**).

### 2.4 Immunothrombotic mechanism and management

Investigation into the thrombotic etiology on day 3 revealed transient aPL positivity: the patient was positive for anticardiolipin IgM (35.26 MPL/mL) and lupus anticoagulant, while anticardiolipin IgG, IgA, and anti-β2-glycoprotein I antibodies (IgG, IgM, IgA) were negative (**Supplementary Table S2**). The temporal correlation between the “pentasystem” thrombotic event, the identification of antiphospholipid antibodies, and the subsequent therapeutic adjustments is summarized in **Figure 2**.

**Figure 2:**
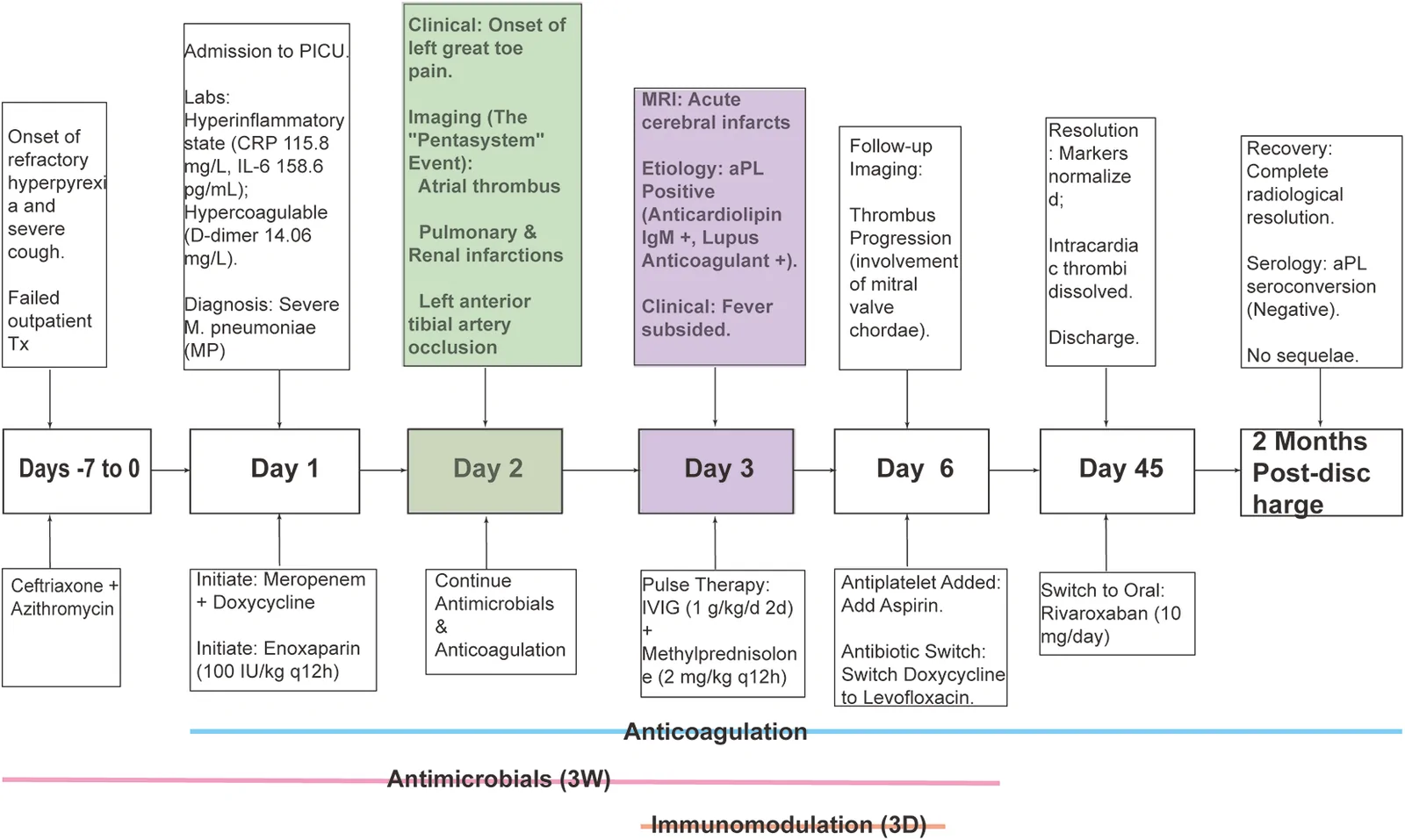
Timeline of clinical course and therapeutic interventions. Data are plotted against hospital days. (Upper Panel) Clinical milestones and diagnostic findings. Transparent shaded vertical bands highlight two critical phases: the “Pentasystem” thrombotic event on Day 2 (green) and the identification of antiphospholipid antibodies on Day 3 (purple). (Lower Panel) Dynamic adjustment of therapeutic regimens. Three distinct color-coded horizontal strips at the bottom represent the duration of antimicrobial, anticoagulant, and immunomodulatory therapies, respectively. Note the switch in antibiotics and the addition of antiplatelet therapy on Day 6 in response to thrombus progression. aPLs, antiphospholipid antibodies; D, day; IVIG, intravenous immunoglobulin; MP, *Mycoplasma pneumoniae*; PICU, pediatric intensive care unit; W, week.

Given the life-threatening thrombotic burden, the regimen was promptly intensified to include immunomodulation with intravenous immunoglobulin (IVIG, 1 g/kg/day for 2 days) and methylprednisolone (2 mg/kg/dose q12h). Although fever subsided by day 3, follow-up imaging on day 6 showed thrombus progression involving the mitral valve chordae and persistent lower limb occlusion (**Supplementary Figure S5**). Consequently, antiplatelet therapy (Aspirin) was added to the anticoagulation regimen, and antimicrobials were adjusted to Meropenem plus Levofloxacin (as illustrated in the lower panel of **Figure 2**). Bronchoscopy with lavage was performed to manage severe airway involvement.

### 2.5 Outcome and follow-up

The patient responded to this multimodal strategy and was discharged on February 2, 2026 (Day 45 of hospitalization). At discharge, inflammatory markers had normalized, brain MRI showed improvement in infarcts, and echocardiography confirmed the complete dissolution of intracardiac thrombi. Secondary prevention was maintained with oral Rivaroxaban (10 mg/day).

At the two-month follow-up, a complete clinical and radiological recovery was observed. Antiphospholipid antibodies seroconverted to negative (**Supplementary Table S2**). Repeat imaging demonstrated near-complete resolution of pulmonary infiltrates and renal infarcts, and lower limb color Doppler ultrasound showed no evidence of vascular embolism, with no residual thromboembolism or neurological sequelae.

This case of severe pentasystem embolism prompted us to systematically review the literature to contextualize its features, particularly the role of antiphospholipid antibodies, within the spectrum of thromboembolic complications in pediatric severe M. *pneumoniae* pneumonia.

## 3 Literature review

### 3.1 Search strategy and study selection

A systematic search was executed across PubMed, Web of Science, CNKI, and the Chinese Medical Journal Full-text Database for literature published from January 2020 through December 2025. The search strategy for PubMed was: (“Pediatrics”[MeSH] OR “Child”[MeSH] OR pediatric[tiab] OR child[tiab] OR children[tiab]) AND (“Mycoplasma pneumoniae”[MeSH] OR “Mycoplasma pneumoniae”[tiab]) AND (“Embolism”[MeSH] OR “Thrombosis”[MeSH] OR “Venous Thromboembolism”[MeSH] OR embolism[tiab] OR thrombosis[tiab] OR thromboembolism[tiab] OR thrombus[tiab]) with date restriction set to 2020:2025[pdat]. The search strategy for Web of Science was: pediatric OR child OR children (Topic) AND “Mycoplasma pneumoniae” (Topic) AND embolism OR thrombosis OR thromboembolism OR thrombus (Topic) with the timespan restricted to PY=(2020-2025). For CNKI, the search terms were: 儿童 (children) AND 支原体肺炎 (Mycoplasma pneumoniae pneumonia) AND 栓塞 (embolism), using fuzzy matching, with the publication date restricted to 2020–2025. For the Chinese Medical Journal Full-text Database, the search was: ((儿童[Children]) AND (支原体肺炎[Mycoplasma pneumoniae pneumonia])) AND (栓塞[Embolism]), with the publication date restricted to January 1, 2020 through December 31, 2025.

Studies were included if they met the following criteria: (1) age <18 years; (2) confirmed MPP diagnosis; (3) imaging-confirmed thrombotic events; and (4) published between 2020 and 2025. Exclusion criteria were: (1) duplicate reports from overlapping cohorts; (2) incomplete clinical data; and (3) publications in languages other than English or Chinese.

Two independent reviewers performed screening of titles, abstracts, and full texts, with disagreements resolved by consensus or a third reviewer. Data were extracted independently using a standardized form and cross-checked for accuracy. Study quality was assessed using a custom-modified checklist derived from the Joanna Briggs Institute (JBI) critical appraisal checklist for case series, focusing on four domains: case definition clarity, completeness of clinical data, thrombotic event reporting, and outcome documentation. Quality scores were not used for exclusion; rather, they informed evidence interpretation in the Discussion.

Screening of titles, abstracts, and full texts yielded 19 non-redundant studies (17 from China, 2 from the USA). To prevent data duplication, only the most comprehensive report was selected when overlapping cohorts were identified. The study selection process is illustrated in the PRISMA flow diagram (**Figure 3**). The final pooled cohort comprised 199 pediatric patients with severe M. *pneumoniae* pneumonia (SMPP) complicated by thromboembolic events.

**Figure 3:**
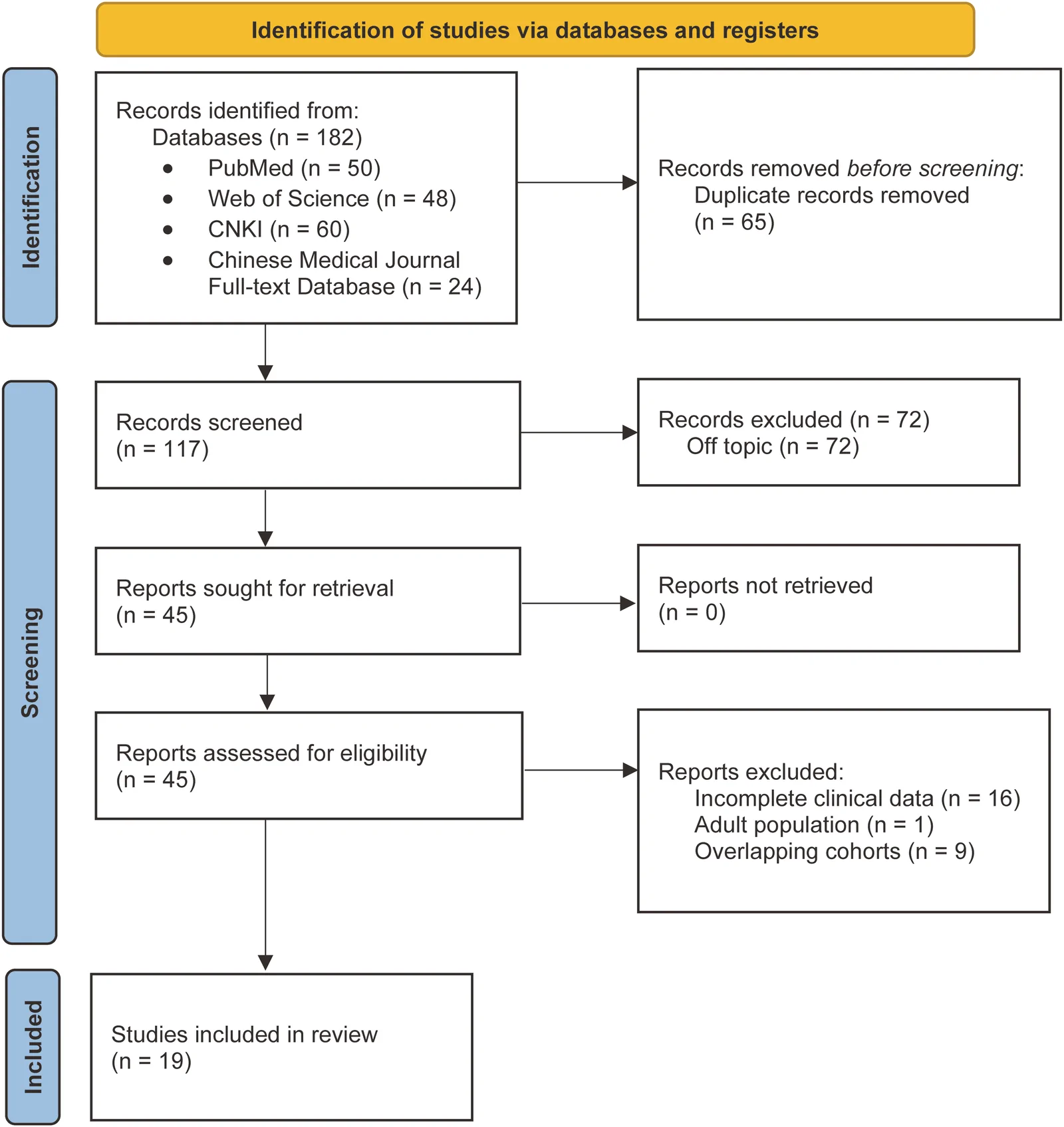
PRISMA flow diagram of the study selection process. A total of 182 records were identified from four databases. After duplicate removal, 117 records were screened by title and abstract, of which 72 were excluded. The remaining 45 full-text reports were assessed for eligibility; 26 were excluded for incomplete data (n = 16), adult population (n = 1), or overlapping cohorts (n = 9). Finally, 19 studies were included.

Statistical analysis. Descriptive statistics were used. Categorical variables are presented as n (%), continuous variables as median (range).

### 3.2 Baseline characteristics

Baseline data are detailed in **Table 1**. The cohort demonstrated a male preponderance (107/199, 53.8%) with a median age of 7.5 years. Systemic inflammatory markers—specifically D-dimer, C-reactive protein (CRP), and lactate dehydrogenase (LDH)—were markedly elevated across the patient population.

**Table 1:** Summary of baseline characteristics, laboratory profiles, and thrombotic manifestations in the included studies (n=19).

Study (author, year, country)	No. of patients	Male, n (%)	Age, median (range)	Elevated inflammatory markers	aPLs testing (positive)	Reported embolism locations (n)	Multi-system embolism (≥2 sites)
Chen et al., 2023, China* (6)	49	27 (55.1)	7.9 yrs	D-dimer, CRP, LDH, ESR	39 t; 26 pos (66.7%)	PE 33, PVT 5, CA 8, CI 9, DVT 4, SI 4, CT & SMA 1	13/49 (26.5%); 2 sites 7, 3 sites 6
Chen et al., 2023, China‡ (22)	4	4 (100)	7 yrs (3–11)	D-dimer, CRP	--	PE 2, PE+DVT 2	2/4 (50.0%); 2 sites 2
Cheng et al., 2024, China (23)	1	0 (0)	6 yrs	D-dimer, CRP	1 t; 1 pos (100%)	PE 1	0/1 (0%)
Fan et al., 2025, China (5)	11	5 (45.5)	8 yrs (5–14)	D-dimer, CRP	--	PE 11	0/11 (0%)
Hahn et al., 2021, USA (17)	1	1 (100)	10 yrs	CRP, LDH, D-dimer, Ferritin	Negative	PE+CA+SI+DVT 1	1/1 (100%); 4 sites 1
Han et al., 2022, China (2)	16	6 (37.5)	7.13 yrs (2–12)	D-dimer, CRP, LDH	--	PE 7, CI 1, SI 1, CA 1, PE+CA 2, PE+IJV 1, CA+PAE 1, ICA+CA 1, ICA+CI 1	6/16 (37.5%); 2 sites 6
Jin et al., 2024, China (24)	5	2 (40.0)	6.8 yrs (5–9)	CRP, LDH, D-dimer, IL-6, Ferritin	--	PE 4, CI 1	0/5 (0%)
Li et al., 2025, China (16)	1	1 (100)	6 yrs	CRP, LDH, D-dimer	--	PE+CA 1	1/1 (100%); 2 sites 1
Liang & Liu, 2024, China (21)	1	1 (100)	7 yrs	CRP, D-dimer, LDH	Pos (aCL+, aβ2GPI+)	PE 1	0/1 (0%)
Saleem & Berman, 2020, USA (20)	2	0 (0)	13 yrs	Not reported	2 t; 2 pos (100%)	PE+DVT 2	2/2 (100%); 2 sites 2
Sheng et al., 2021, China (10)	7	4 (57.1)	8 yrs (6–11)	CRP, D-dimer	7 t; 6 pos (85.7%)	PE 6, PE+DVT 1	1/7 (14.3%); 2 sites 1
Wang et al., 2021, China (15)	1	0 (0)	5 yrs	D-dimer, CRP, Fibrinogen	Pos (ACA IgM+)	CA+CI 1	1/1 (100%); 2 sites 1
Wang et al., 2024, China (7)	16	7 (43.8)	8.2 yrs (5.2–11.4)	D-dimer, CRP, LDH	4 t; 2 pos (50%)	PE+SI 1, PE 14, PE+DVT 1	2/16 (12.5%); 2 sites 2
Wang et al., 2025, China (11)	4	2 (50.0)	5 yrs (4–7)	CRP, ESR, D-dimer, IL-6	--	CI 2, CI+CA+PAE 1, CI+PAE 1	2/4 (50.0%); 2 sites 1, 3 sites 1
Xiaoqian & Hemin, 2025, China (25)	1	1 (100)	10 yrs	CRP, D-dimer	--	PE 1	0/1 (0%)
Xie et al., 2024, China (26)	62	34 (54.8)	7.5 yrs (3–12)	D-dimer, IL-6, NLR, CRP, LDH	--	PE 46, CA 7, SI 4, CI 1, RE 1, SVC 1, IVC 1	13/62 (21.0%); 2 sites 13
Yang Juan et al., 2025, China (13)	15	10 (66.7)	8.0 yrs	D-dimer, CRP, LDH	8 t; 6 pos (75%)	CA+CI+PE 9, CA 5, CA+CI+PE+DVT 1	10/15 (66.7%); 3 sites 9, 4 sites 1
Zhang et al., 2025, China† (27)	1	1 (100)	9 yrs	D-dimer, CRP	--	PE 1	0/1 (0%)
Zhang et al., 2025, China§ (28)	1	1 (100)	3.7 yrs	D-dimer	Negative	PAE 1	0/1 (0%)
Total/Pooled	199	107 (53.8)	--	-–	65 t; 45 pos (69.2%)	--	54/199 (27.1%); 2 sites 36, 3 sites 16, 4 sites 2

Data presented in this table are summarized from the 19 studies included in the systematic review (see Section 3).

aβ2GPI, anti-β2-glycoprotein I antibody; ACA/aCL, anticardiolipin antibody; aPLs, antiphospholipid antibodies; CA, cardiac thrombosis; CI, cerebral infarction; CRP, C-reactive protein; CT & SMA, celiac trunk and superior mesenteric artery embolism; DVT, deep vein thrombosis; ESR, erythrocyte sedimentation rate; ICA, internal carotid artery; IJV, internal jugular vein thrombosis; IVC, inferior vena cava thrombosis; LDH, lactate dehydrogenase; NLR, neutrophil-to-lymphocyte ratio; PAE, peripheral arterial embolism; PE, pulmonary embolism; pos, positive; PVT, pulmonary vein thrombosis; RE, renal embolism; SI, splenic infarction; SVC, superior vena cava thrombosis; t, tested.

Symbols: “--” indicates that the corresponding information was not reported in the original study. Study Identification: *, Chen et al., 2023 refers to the study from Beijing Children’s Hospital; ‡, Chen et al., 2023 refers to the study from Shanghai Children’s Hospital; †, Zhang et al., 2025 refers to the study from Dalian Women and Children’s Medical Group; §, Zhang et al., 2025 refers to the study from Hebei Provincial Hospital of Traditional Chinese Medicine.

### 3.3 Antiphospholipid antibody profile

Testing for antiphospholipid antibodies (aPLs) was documented in 9 of the 19 studies (47.4%), comprising a subset of 65 patients (32.7% of the total cohort). Within this tested group, 45 patients were aPLs-positive, corresponding to a positivity rate of 69.2% (**Table 1**).

### 3.4 Spectrum of thrombotic manifestations

Thrombosis affected diverse vascular beds, with pulmonary embolism (PE) being the primary manifestation. Multi-system involvement (≥2 vascular territories) was identified in 54 patients (27.1%) (**Table 1**). Stratification of these multi-system cases revealed involvement of two sites in 36 patients, three sites in 16 patients, and four sites in 2 patients.

### 3.5 Treatment regimens and clinical outcomes

Anticoagulation therapy was universally administered (199/199, 100%; **Table 2**). Immunosuppressive therapy, predominantly corticosteroids, was a standard intervention, particularly within Chinese cohorts, whereas intravenous immunoglobulin (IVIG) usage was explicitly reported in only three studies (15.8%). Invasive surgical or interventional procedures were reserved for refractory cases. Despite the severity of thrombotic complications, outcomes were generally favorable, with most studies reporting full or substantial recovery. Documented sequelae included bronchiolitis obliterans (n=4), neurological deficits (n=3), and two fatalities.

**Table 2:** Summary of treatment strategies and clinical outcomes.

Study (author, year, country)	N	Anticoagulation (n)	Immunosuppressive therapy (n)	Surgical intervention	Clinical outcome
Chen et al., 2023, China* (6)	49	AC (48), AC+LT (1)	--	1 resection (cardiac mass)	Favorable; 1 case with hemiplegia
Chen et al., 2023, China‡ (22)	4	AC (4)	--	--	All recovered
Cheng et al., 2024, China (23)	1	AC (1)	CS (1)	--	Full recovery; aPLs negated
Fan et al., 2025, China (5)	11	AC (11)	--	--	7 resolved; 4 developed BO
Hahn et al., 2021, USA (17)	1	AC (1)	Not used	--	Full recovery; no sequelae
Han et al., 2022, China (2)	16	AC+LT (2), AC+AP (1), AC (13)	CS (16)	--	Favorable; mild neuro sequelae
Jin et al., 2024, China (24)	5	AC+AP (5)	CS (5); IVIG (3)	--	Resolved; 1 with tremor/motor sequelae
Li et al., 2025, China (16)	1	AC (1)	CS (1)	Bowel resection (necrosis)	Good; PE resolved
Liang & Liu, 2024, China (21)	1	AC (1)	CS+IVIG	--	Good
Saleem & Berman, 2020, USA (20)	2	AC (2)	--	--	Full recovery after antibiotics & AC
Sheng et al., 2021, China (10)	7	AC (7)	--	5 pulmonary infarct resections	2 deaths
Wang et al., 2021, China (15)	1	AC+LT (1)	--	--	Good; intracardiac embolism resolved
Wang et al., 2024, China (7)	16	AC (16)	CS (16)	--	Favorable; no recurrence
Wang et al., 2025, China (11)	4	AC (4)	CS (2), CS+IVIG (2)	--	Good recovery
Xiaoqian & Hemin, 2025, China (25)	1	AC (1)	CS (1)	--	Good; PE resolved
Xie et al., 2024, China (26)	62	AC (62)	CS (62)	--	--
Yang Juan et al., 2025, China (13)	15	AC (11), AC+LT (4)	--	Cardiac thrombectomy (5)	Good recovery
Zhang et al., 2025, China† (27)	1	AC (1)	CS (1)	--	Complete recovery
Zhang et al., 2025, China§ (28)	1	AC+LT (1)	Not used	Interventional thrombectomy	Good; functional recovery
Total/Summary	199	199/199 (100.0%)	Common (in Chinese studies)	Selected severe cases	Variable; majority favorable

AC, anticoagulation; LT, thrombolysis; AP, antiplatelet; CS, corticosteroids; IVIG, intravenous immunoglobulin; BO, bronchiolitis obliterans; PE, pulmonary embolism.

Symbol: “--” indicates that the corresponding information was not reported in the original study.

See **Table 1** for footnote designations (*, ‡, †, §).

The treatment strategies and clinical outcomes summarized in this table are derived from the 19 studies included in our systematic review (see Section 3).

## 4 Discussion

### 4.1 Unique phenotype and epidemiological context

This case report delineates an extreme phenotype of pentasystem embolism in a child with severe *Mycoplasma pneumoniae* pneumonia (SMPP), representing a degree of thrombotic burden rarely documented in existing literature. Coupled with our systematic review of 199 pediatric cases, this study substantiates that thromboembolic complications are not merely isolated incidents but a pervasive threat in SMPP. Our analysis reveals that 27.1% (54/199) of thrombotic cases involved multi-system embolism, defining a severe disease subset. This high prevalence of disseminated involvement provides essential context for the broader thrombotic risk in SMPP. For instance, the large cohort study by Xie et al. reported that 35.4% (62/175) of hospitalized SMPP children experienced thromboembolic events, supporting thrombosis as a frequent rather than rare complication (26). Within this at-risk population, our review clarifies that a significant proportion progresses to multi-system disease, posing a severe management challenge. While pulmonary embolism remains the predominant initial manifestation consistent with the pulmonary tropism of MP (6, 10, 22, 29), our findings emphasize vigilance for extrapulmonary events.

### 4.2 Pathophysiology: the “Dual-Hit” mechanism

The pathogenesis of such extensive thrombosis aligns with a “dual-hit” hypothesis. The initial “hit” entails a robust systemic inflammatory response. As detailed in recent reviews of MP-associated hypercoagulability, MP infection triggers a “cytokine storm” (evidenced by our patient’s markedly elevated CRP and IL-6), which directly induces endothelial injury and upregulates tissue factor expression, shifting the vascular bed to a prothrombotic state (3, 18, 30–32). This link between inflammation and coagulation is well-established in severe MP infections (2, 27).

The subsequent “second hit” likely involves the emergence of acquired, transient antiphospholipid antibodies (aPLs). Our patient exhibited positive anticardiolipin IgM and lupus anticoagulant during the acute phase. Our pooled analysis supports this mechanism, revealing a high aPLs positivity rate (69.2%) among tested patients. These antibodies may serve as a potential risk-amplifying biomarker (20, 21), though their independent pathogenic contribution beyond the baseline inflammatory milieu remains uncertain. The concomitant weak ANA positivity further implies that MP can trigger a broader, albeit transient, autoimmune phenomenon (6).

Several caveats warrant consideration. First, the temporal association we observed does not equate to a causal relationship. The pronounced hyperinflammatory response—characterized by marked elevations in IL-6, CRP, and D-dimer—alone may be sufficient to explain the extensive thrombotic manifestations, given that cytokine-driven endothelial injury, tissue factor upregulation, platelet activation, and immobilization are all recognized prothrombotic mechanisms in severe infections (30–32). Second, the aPLs detected in this setting could represent an epiphenomenon of polyclonal B-cell activation during the acute immune response, rather than a primary thrombotic driver (33, 34). Thus, while transient aPL positivity may retain utility as a clinical biomarker, its independent pathogenic contribution beyond the baseline inflammatory milieu remains uncertain. Similar immunothrombotic mechanisms have also been reported in other infectious settings (30, 31).

### 4.3 Predictive thresholds for risk stratification

While elevated D-dimer is universal in SMPP, defining a predictive cutoff is crucial for bedside risk stratification. Xie et al. developed a nomogram identifying D-dimer and IL-6 as independent risk factors, supporting a linear correlation with thrombotic risk (26). Previous studies have indicated that D-dimer levels exceeding 5.0 mg/L, or even 11.1 mg/L as identified by Liu et al., serve as strong predictors for thrombosis (26, 35). Similarly, Han et al. noted that D-dimer levels in embolus groups were markedly higher than in non-embolus SMPP controls (2). In our patient, the D-dimer peak (14.06 mg/L) far exceeded these “red flag” thresholds, consistent with the severe pentasystem involvement we observed. Consequently, we suggest that D-dimer values >5–10 times the upper limit of normal in SMPP should prompt vascular imaging, even in the absence of overt ischemic symptoms.

### 4.4 The diagnostic gap regarding aPLs

A critical disparity exists between the pathophysiological role of aPLs and current clinical practice. Despite the high aPLs positivity in tested cohorts, our review identified that only 9 of 19 studies (47.4%) reported aPLs results, covering merely 32.7% of the total patient pool. This suggests that in over two-thirds of reported cases, this pivotal and potentially modifiable risk factor was overlooked. This under-detection likely stems from insufficient awareness of infection-induced autoimmune phenomena (3). Given that aPLs positivity correlates with thrombotic severity (36), we strongly advocate incorporating aPL testing into the standard workup for SMPP patients with suspected thrombosis.

### 4.5 Prognosis and duration of anticoagulation

Crucially, the nature of SMPP-associated aPLs differs fundamentally from primary antiphospholipid syndrome (APS). Unlike APS, which requires persistent positivity (37), MP-associated aPLs are typically transient and infection-driven, often seroconverting to negative upon resolution of inflammation (7, 17). This “transient” characteristic likely explains why, despite severe presentations, the majority of patients achieve favorable outcomes with low mortality (2/199), particularly when managed with combined anticoagulation and immunomodulation (e.g., corticosteroids, IVIG) (see **Table 2**). Our patient’s favorable response to this combined approach underscores the importance of early immunomodulation in aPLs-positive cases and should reassure clinicians about the potentially reversible nature of this acquired autoimmune thrombophilia. Clinicians should therefore refrain from labeling acute MP infection-associated aPL positivity as “antiphospholipid syndrome,” and should instead characterize it as transient, infection-associated aPL positivity. In particular, isolated transient IgM positivity during acute infection should not be equated with pathogenic APS-like autoimmunity. Notably, although most patients recover well, fibrotic sequelae such as bronchiolitis obliterans have been reported in some SMPP cases, with p38-related pathways implicated in pulmonary fibrosis (38).

Given this transient and reversible nature, the optimal duration of anticoagulation differs from unprovoked thrombosis. Based on our review and case experience, we propose a “biomarker-guided” cessation strategy. Anticoagulation may be safely discontinued when three criteria are met: (1) full clinical recovery, (2) radiological resolution of thrombi, which may involve angiogenic repair mechanisms (39), and (3) seroconversion of aPLs to negative (20, 25). Our patient followed this protocol, stopping therapy after 3 months upon seronegativity and thrombus resolution, with no recurrence. This contrasts with the lifelong anticoagulation often required for primary APS.

### 4.6 Limitations

Several limitations of this study must be acknowledged. First, the systematic review relied predominantly on retrospective, single-center case series, which inherently introduces selection and reporting biases. Second, a distinct geographic clustering was observed, with the vast majority of included studies (17 of 19) originating from China. While this distribution may limit the generalizability of our findings to non-Asian populations, it likely reflects a true epidemiological signal related to the high prevalence of macrolide-resistant M. pneumoniae (MRMP) in East Asia. This resistance pattern is associated with stronger inflammatory responses and potentially higher thrombotic risks (7), underscoring the need for global vigilance as MRMP strains spread worldwide. Third, the heterogeneity in aPLs testing—reported in less than half of the included articles (47.4%)—hinders a definitive assessment of the true prevalence of infection-induced autoimmunity. Fourth, the observational design precludes causal inference on aPLs in thrombosis; whether these antibodies are pathogenic or bystanders remains undetermined. Fifth, aPL testing in the pooled cohort was limited—only 32.7% underwent testing, and panels often omitted anti-β2GPI. This incomplete profiling limits generalizability of prevalence estimates. Finally, while our case provides a vivid illustration of the “dual-hit” mechanism, its extreme “pentasystem” phenotype represents the severe end of the clinical spectrum and may not reflect the typical course of SMPP-associated thrombosis.

## 5 Conclusion

Take-Home Messages

- Severe M. pneumoniae pneumonia in children can cause multi-vascular-bed thromboembolism; markedly elevated D-dimer should prompt vascular imaging.
- Transient aPL positivity is a risk-amplifying biomarker, not antiphospholipid syndrome during acute infection.
- Combined anticoagulation and immunomodulation improve outcomes; cessation can be guided by clinical recovery, thrombus resolution, and aPL seroconversion.

In conclusion, this study provides clinical evidence consistent with a “dual-hit” mechanism—synergistic inflammation and transient autoimmunity—associated with extensive multi-system embolism in children with SMPP. To navigate this clinical challenge, we propose a stratified management triad: (1) Precision Screening utilizing D-dimer thresholds (>5.0 mg/L) and aPLs profiling for early risk stratification; (2) Targeted Antimicrobial Therapy addressing potential macrolide resistance; and (3) Combined Immunothrombotic Therapy, integrating anticoagulation with timely immunomodulation to mitigate the autoimmune “second hit.” Ultimately, this multimodal framework offers a practical roadmap for clinicians to timely recognize and reverse this critical condition and may help transform a life-threatening emergency into a manageable, reversible event. Future prospective studies are warranted to validate these findings and optimize management strategies.

## Data Availability

The original contributions presented in the study are included in the article/**Supplementary Material**. Further inquiries can be directed to the corresponding authors.
