# Supplementary material for: Pentasystem embolism associated with transient antiphospholipid antibodies in severe M. pneumoniae pneumonia: a “Dual-Hit” case report and systematic review
Source: Front Immunol. 2026 Sep 30;17:1845246. doi: 10.3389/fimmu.2026.1845246 (PMC13661489; doi:10.3389/fimmu.2026.1845246)
Supplement: Supplementary file 1 [file DataSheet1.docx]

**Supplementary Figure S1. Original metagenomic next-generation sequencing (mNGS) report of the peripheral blood sample.**

The report confirms the detection of Mycoplasma pneumoniae. The key data points are as follows: Detected Pathogen: *Mycoplasma pneumoniae* (肺炎支原体)

(Note: The original report is in Chinese; key identifiers have been translated above.)

**Supplementary Figure S2. Multisite thromboembolism and visceral infarction on contrast-enhanced CT**

(A) Coronal reformatted chest CT reveals a thrombus (red arrow) extending from the right inferior pulmonary vein into the left atrium. (B) Axial chest CT provides a cross-sectional confirmation of the same intracardiac thrombus (red arrow). (C) Axial contrast-enhanced chest CT (mediastinal window) demonstrates a filling defect (red arrow) within the right inferior pulmonary vein, adjacent to a large, hypoattenuating area of consolidation (yellow arrow) in the right lower lobe, suggestive of pulmonary infarction. (D, E) Axial abdominal CT images display wedge-shaped non-enhancing hypodensities (red arrows) in the renal parenchyma, consistent with bilateral acute renal infarcts.

**Supplementary Figure S3. Ultrasonographic evaluation of cardio-peripheral thromboembolism.**

(A, B) Transthoracic echocardiography (TTE) visualizes an echogenic mass (red arrows) projecting into the left atrium from the orifice of the right pulmonary vein. (C) Color Doppler ultrasound of the contralateral (right) anterior tibial artery demonstrates a patent lumen with normal flow signals. (D) In contrast, the left anterior tibial artery shows a complete flow void (red arrow), indicative of acute arterial occlusion.

**Supplementary Figure S4. Cranial MRI (DWI sequence) demonstrating multi-focal acute ischemic lesions.**

(A) Axial Diffusion-Weighted Imaging (DWI) reveals two discrete hyperintense foci (red arrows) within the right cerebellar hemisphere. (B) A single focus of restricted diffusion (red arrow) is identified in the left parieto-occipital white matter. (C, D) Additional hyperintensities (red arrows) are visualized in the left centrum semiovale and left frontal subcortical region, respectively, consistent with an embolic pattern.

**Supplementary Figure S5. Follow-up ultrasonographic assessment of thrombotic evolution (Day 6).**

(A, B) Transthoracic echocardiography (TTE) depicts the progression of the intracardiac thrombus (red arrows), which now involves the mitral valve apparatus, specifically adhering to the chordae tendineae. (C) Color Doppler ultrasound of the left anterior tibial artery confirms a persistent flow void (red arrow), signifying unresolved arterial occlusion despite anticoagulation.
