# Supplementary material for: Pentasystem embolism associated with transient antiphospholipid antibodies in severe M. pneumoniae pneumonia: a “Dual-Hit” case report and systematic review
Source: Front Immunol. 2026 Sep 30;17:1845246. doi: 10.3389/fimmu.2026.1845246 (PMC13661489; doi:10.3389/fimmu.2026.1845246)
Supplement: Supplementary file 2 [file Table1.docx]

**Supplementary Table S1. Targeted next-generation sequencing (tNGS) results of blood specimens**

| Pathogen | Type | Sequence Reads | Relative Abundance | Confidence |
| --- | --- | --- | --- | --- |
| *Mycoplasma pneumoniae* | Gram-negative | 789 | 86.48% | 99% |

Note: Metagenomic next-generation sequencing (mNGS) of blood specimens revealed no other pathogens.
