# Supplementary material for: Pentasystem embolism associated with transient antiphospholipid antibodies in severe M. pneumoniae pneumonia: a “Dual-Hit” case report and systematic review
Source: Front Immunol. 2026 Sep 30;17:1845246. doi: 10.3389/fimmu.2026.1845246 (PMC13661489; doi:10.3389/fimmu.2026.1845246)
Supplement: Supplementary file 3 [file Table2.docx]

**Supplementary Table S2. Profile of antiphospholipid antibodies (aPLs) in the index patient**

| Antibody / Assay | Result (Day 3 of hospitalization) | Reference Range | Interpretation | Repeat Testing (March 7, 2026) |
| --- | --- | --- | --- | --- |
| Anticardiolipin antibodies |  |  |  |  |
| aCL IgA | < 2.00 APL/mL | < 20.00 APL/mL | Negative | Negative |
| aCL IgM | 35.26 MPL/mL | < 20.00 MPL/mL | Positive | Negative |
| aCL IgG | 3.08 GPL/mL | < 20.00 GPL/mL | Negative | Negative |
| Anti-β2-glycoprotein I antibodies |  |  |  |  |
| Anti-β2GPI IgG | 10 RU/mL | < 20 RU/mL | Negative | Negative |
| Anti-β2GPI IgM | 16 RU/mL | < 20 RU/mL | Negative | Negative |
| Anti-β2GPI IgA | 4 RU/mL | < 20 RU/mL | Negative | Negative |
| Lupus anticoagulant (dRVVT) |  |  |  |  |
| — Screening ratio | 1.50 | < 1.20 | Positive | Negative |
| — Standardized ratio | 1.54 | 0.80–1.20 | Positive | Negative |
| — Confirmatory ratio | 0.97 | < 1.20 | Negative | Negative |

Abbreviation: aCL, anticardiolipin antibodies.
